# Estimating demand for perennial pigeon pea in Malawi using choice experiments

**DOI:** 10.1016/j.ecolecon.2016.09.006

**Published:** 2017-01

**Authors:** Kurt B. Waldman, David L. Ortega, Robert B. Richardson, Sieglinde S. Snapp

**Affiliations:** a Ostrom Workshop in Political Theory and Policy Analysis, Indiana University, USA; b Dept. of Agricultural, Food, and Resource Economics, Michigan State University, USA; c Dept. of Community Sustainability, Michigan State University, USA; d Dept. of Plant, Soil and Microbial Sciences, Michigan State University, USA

**Keywords:** Pigeon pea, Malawi, Perennial, Choice experiment, Farmer preferences, Soil fertility

## Abstract

Perennial crops have numerous ecological and agronomic advantages over their annual counterparts. We estimate discrete choice models to evaluate farmers' preferences for perennial attributes of pigeon pea intercropped with maize in central and southern Malawi. Pigeon pea is a nitrogen-fixing leguminous crop, which has the potential to ameliorate soil fertility problems related to continuous maize cultivation, which are common in Southern Africa. Adoption of annual pigeon pea is relatively low but perennial production of pigeon pea may be more appealing to farmers due to some of the ancillary benefits associated with perenniality. We model perennial production of pigeon pea as a function of the attributes that differ between annual and perennial production: lower labor and seed requirements resulting from a single planting with multiple harvests, enhanced soil fertility and higher levels of biomass production. The primary tradeoff associated with perennial pigeon pea intercropped with maize is competition with maize in subsequent years of production. While maize yield is approximately twice as valuable to farmers as pigeon pea yield, we find positive yet heterogeneous demand for perenniality driven by soil fertility improvements and pigeon pea grain yield.

## 1 Introduction

Population growth and rising consumption are dramatically increasing demands on agriculture and natural resources, which raises challenges for achieving global food security (Foley et al., 2011). Sub-Saharan Africa (SSA) has struggled to achieve food security for myriad reasons including poor quality soils, land degradation, low levels of fertilization, market failure, and poor infrastructure and transportation (de Graaff et al., 2011). The growing population in SSA is putting additional pressure to produce more food on the same amount of land, putting food security even further out of reach (United Nations, Population Division of the Department of Economic and Social Affairs, 2014). The Green Revolution strategies of intensive cultivation through improved crop germplasm and more wide-scale fertilizer use might not be enough to feed 9 billion people in the future (Godfray et al., 2010). A primary reason for this is that smallholder farmers rarely benefit from improved germplasm because they are farming on depleted soils that are often not responsive to fertilizer (Giller et al., 2006, Tittonell et al., 2007).

Declining soil fertility is exacerbated in Southern Africa by wide-scale continuous cultivation of maize, which mines the soil of nutrients and leaves farmers struggling to maintain yields, year after year (Snapp et al., 2010). Incorporating nitrogen-fixing legumes into the cropping systems has the potential to improve soil fertility and mitigate the nutrient mining impact of maize (Snapp and Silim, 2002a, Bezner-Kerr et al., 2007). In addition to providing much needed nitrogen, leguminous crops are high in protein making them valuable nutritionally. One legume with a wide variety of uses is pigeon pea (*Cajanus cajan*). In addition to the nitrogen-fixing properties and protein rich grain, pigeon pea provides a range of byproducts including leaves and stems used for fodder and the dried stems for fuel wood (Simtowe et al., 2010). Despite these benefits, adoption of leguminous crops and particularly pigeon pea in SSA remains low (Snapp and Silim, 2002a).

Low adoption of beneficial crops and improved varieties of crops in Africa may be related to the difficulty in transitioning from low-input, subsistence (extensive) agriculture to high-input, intensive, market-based production (Dingkuhn et al., 2006). In contrast to the types of intensive varieties developed in the Green Revolution, smallholder farmers in Africa may be more risk- averse and seek a diversity of crops and varieties that fit different farming system niches (Altieri, 2002). A diversification strategy that allows farmers to spread short-term risk and develop varieties that adapt to changing climatic conditions might improve the resilience of African smallholder systems (Morton, 2007). Perennial crops are one possible technology for investment in the long-term resilience of such systems.

Perennial crops present numerous tradeoffs to farmers. The primary tradeoff associated with perennial pigeon pea intercropped with maize is competition with maize in subsequent years of production. Since perennial crops yield less than annual crops, efforts to develop perennial wheat have focused on improved grain yield (Cox et al., 2006, Jaikumar et al., 2012, Hayes et al., 2012). However, Adebiyi et al. (2015) found that farmers in Michigan who experimented with perennial wheat described soil and environmental quality as their primary motivations for doing so. An emphasis on yield in a low-input, low-output developing country environment may not capture the tradeoffs perceived by farmers or the broader ecological benefits. According to Snapp et al. (2005), environmental benefits from cover crops predominantly accrue to society as a whole (e.g., erosion control), whereas (opportunity) costs are often local (e.g., planting a perennial crop may mean displacing a higher yielding annual). This has implications for the case of perennial grain crops, where society-level environmental services have been studied but local, farm-level benefits and costs are not known.

The main objective of this study is to assess the tradeoffs involved in annual versus perennial pigeon pea production from the perspective of Malawian smallholder farmers. We quantify the various attributes of interest and estimate demand for individual characteristics of perenniality. By exploring preferences for perennial pigeon pea we also contribute to the literature on farmer preferences regarding sustainable agricultural practices such as farmer awareness (D'Souza et al., 1993) and the long-term investment and payoff dimension (Caviglia-Harris et al., 2003). We also contribute to the literature on using choice experiments to measure smallholder preferences for crop diversity, including plant genetic resources (Birol et al., 2009), agrobiodiversity (Birol et al., 2006), local landraces (Smale et al., 2001) and hybrids (Smale and Olwande, 2014).

## 2 Background on Perennial Crops

Since a perennial crop does not need to be planted every year, less tillage is required which translates into less soil disturbance and erosion and the development of a larger root mass. The large root mass helps retain soil, prevent future erosion, and sequester more carbon since the roots remain in the ground year round. Perennial systems have more soil fauna diversity and natural belowground processes since they have more year round vegetation (Culman et al., 2010). Perennial crops also use less seed inputs since they require reseeding every three to five years as opposed to annual crops (Bell et al., 2008). As a result, the farm labor costs, energy usage, and technological inputs required for yearly tillage is lower in perennial crop systems (Pimentel et al., 2012). The larger root systems are effective at absorbing nutrients and improving water quality by preventing nutrient leaching (Culman et al., 2013). Perennial plant root structures reach more deeply into the soil and hold more soil water (Glover et al., 2010). This makes perennial crops potentially less risky in low rainfall environments, more resistant to flooding, and more resilient to climate variability.

In low-input, low-output smallholder farming systems that are common in developing countries, the tradeoffs associated with perennial as opposed to annual production are less clear. Farmers in developing countries face numerous production constraints such as labor bottlenecks at planting and harvesting time and lack of capital for purchased inputs. The risk of crop failure is higher when agricultural production takes place in more marginal environments, and where farmers face a high likelihood of depredation from both wild and domesticated animals. Since farmers lack capital to purchase farm inputs they potentially value the ecological advantages such as the soil fertility improvement or increased biomass for fodder, mulch or fuel wood more than a farmer in a developed country. There is emerging evidence that smallholder farmers value perennials, based on studies of agroforestry and semi-perennial cropping systems in Southern and East Africa (Faße and Grote, 2013, Orr et al., 2014, Snapp et al., 2010); however perennial traits of food crops have not been previously investigated.

Pigeon pea provides a unique opportunity to explore perennial traits in a food crop. Recommended production practices for pigeon pea in Malawi are consistent with agronomic recommendations throughout the region, where pigeon pea is treated as an annual crop (Malawi ministry of Agriculture, 2012, Snapp et al., 2003). However, smallholder farmers in Africa and beyond are known to ‘ratoon’ pigeon pea shrubs, which involves cutting back branches after the grain is harvested and then taking a second or even third crop from the regrowth (Tayo, 1985). This treats the pigeon pea shrub as a perennial, and many varieties of pigeon pea have the genetic potential to express perennial traits. Managing pigeon pea as a perennial crop is an understudied subject, and the vast majority of genetic improvement in pigeon pea has focused on developing shorter duration varieties for annual production (Lawn, 1989). There are exceptions, for example the potential for multiple harvests through ratooning of short-duration genotypes was the topic of a few studies carried out in the late 1980s (Chauhan et al., 1987). The existence of both annual and perennial forms of pigeon pea production provides a unique opportunity to explore with farmers the valuation associated with perennial attributes of a food crop.

Crop breeding efforts in Africa have emulated the successes of the Green Revolution by developing high yielding and input responsive germplasm as opposed to developing varieties that integrate traditional crop characteristics that remain essential for farmers (Dingkuhn et al., 2006). Cereal crops common in Africa, such as maize, sorghum and millet have generally been bred for intensive traits such as shorter stature, early-maturation, pest and disease-resistance, input-responsiveness, and the production of multiple crops per year (Stoop et al., 2002). Aside from appreciation of yield, farmers' preferences vary across Sub-Saharan Africa and include non-market criteria such as environmental adaptation (to low-input systems and heterogeneous environments), plant architecture (Isaacs et al., 2016, vom Brocke et al., 2010, Voss, 1992), cooking qualities (Demont et al., 2012), and other consumption properties (Waldman et al., 2014, Ortega et al., 2016). As such we estimate farmer demand for perenniality in the context of perennial pigeon pea production in Malawi.

## 3 Methods

### 3.1 Study Area and Sampling

**Fig. 1:**
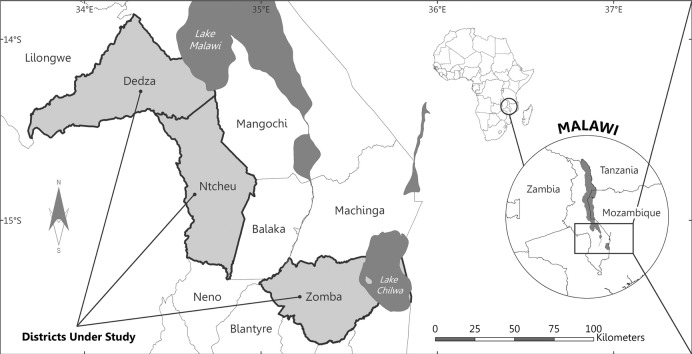
Study area.

The data used in this study are derived from farm household surveys conducted in three districts in Malawi's Central and Southern Regions: Dedza, Ntcheu and Zomba. These districts are highlighted in Fig. 1.

Dedza district is located south of the capital, Lilongwe, has total land area of 3570 km^2^ and has a population of 624,445 according to the 2008 Malawi population Census. Ntcheu district, located to the south of Dedza district, covers an area of 2500 km^2^ and has a population of 471,589. Zomba district, located in Southern region, has a total land area of 1939 km^2^ and a population of 579,639. The respective average population density of Dedza, Ntcheu, and Zomba districts is 175, 189, and 299 persons per square kilometer, the majority living in rural areas. Located between − 14.17 and − 15.17 degrees latitude and with an elevation difference ranging up to 1600 m above sea level, the study area covers various agro-ecological and climatic zones. Rain-fed agriculture predominates in this area, dependent on a single rainy season between November and March. Additionally, these three districts exhibit different patterns of participation in legume and labor markets, as well as different levels of economic development. The study sites include areas where agriculture extension and development projects have been actively promoting legume production through workshops and other outreach efforts, although not specifically pigeon pea.

Our sample consists of farmers from 488 village households that were interviewed in September and October 2014. A multistage sampling approach within each district was used to form the survey sample. In the first stage, we selected four Extension Planning Areas (EPAs) that were dependent on legume production. In the second stage we randomly selected two sections from each EPA where we worked with Agriculture Extension Development Officers (AEDOs) to randomly sample approximately 20 farmers from village rosters in each section. Where village rosters were not available through the section offices, we worked with village leaders to draw a random sample of farmers within each section. After eliminating observations with missing data, our final sample consists of 162, 165, and 161 farm households from Dedza, Ntcheu and Zomba districts, respectively.

### 3.2 Modeling Preferences for Perenniality Using Choice Experiments

We use choice experiments to study farmers' preferences for attributes of a perennial pigeon pea crop. Choice experiments measure the stated preferences of participants as opposed to revealed preferences that come from observed market transactions. Choice modeling is based on Lancastrian consumer theory (Lancaster, 1966) and is used to estimate the marginal value of various attributes of a good. Choice experiments are especially useful when an observed transaction of a good does not occur since they are based on hypothetical choice sets and can thus be used to estimate demand for new products or technologies (Lusk and Shogren, 2007).

Choice experiments have been used in a wide variety of international agricultural and environmental development contexts. For example Roessler et al. (2008) use choice experiments to assess farmers' preferences for pig breeding traits in different production systems in Vietnam; Birol et al. (2012) estimate Filipino farmer's willingness to pay for Bt maize seed; Ruto et al. (2008) evaluate Kenyan cattle producer and trader preferences for indigenous breeds in the pastoral livestock market; Ortega et al. (2014) examine Chinese aquaculture farmer's willingness to adopt good agricultural practices; and Ward et al. (2014) measure Indian farmer preferences for drought tolerant rice.

We design a choice experiment to evaluate preferences for perennial pigeon pea. In our analysis, farmers are assumed to maximize the utility derived from their cropping decision. More formally, we postulate that farmer *n* faces *K* alternatives contained in choice set *ψ*. We define an underlying latent variable *V*_*njs*_^⁎^ that denotes the value function associated with farmer *n* choosing option *j* ∈ *ψ*  during choice task *s*. Farmer *n* will choose alternative *j* so long as *V*_*njs*_^⁎^ > *V*_*nks*_^⁎^ ∀ *k* ≠ *j*. Indirect utility *V*_*njs*_^⁎^ is not directly observed; what is observed is the actual utility maximizing choice *V*_*njs*_, where

(1) $$V_{\mathrm{njs}}=\left\{\begin{array}{c} 1\\ 0 \end{array}\right)\begin{array}{c} \text{if}\;V_{\mathrm{njs}}^{*}=\max\left(V_{n1s}^{*},V_{n2s}^{*},\ldots ,V_{\mathrm{nKs}}^{*}\right)\\ \text{Otherwise} \end{array}.$$

Following standard practice, indirect utility is assumed linear, ensuring that marginal utility is strictly monotonic in the specified attributes and yields corner solutions in which only one choice is selected (Useche et al., 2013). We can therefore write farmer *n*’'s utility function as

(2) $$V_{\mathrm{njs}}^{*}=X_{\mathrm{njs}}^{\prime }\beta +\varepsilon_{\mathrm{njs}}$$

where *X*_*njs*_′ is a vector of characteristics of each choice for the *j*th alternative, *β* is a vector of preference parameters (i.e., a vector of weights mapping attribute levels into utility), and *ε*_*njs*_ is a stochastic component of utility that is independently and identically distributed (iid) across individuals and alternative choices, and takes a predetermined (Gumbel or extreme value type I) distribution. This stochastic component of utility implies that predictions cannot be made with certainty and captures unobserved variations in tastes as well as errors in farmer's perceptions and optimization.

Because farmers are a heterogeneous group, their preferences for cropping system characteristics may also be heterogeneous. A common method of evaluating preference heterogeneity is the estimation of a random parameters logit (RPL) model, also called a mixed logit. Following the RPL specification in Train (2003), the probability that individual *n* chooses alternative *j* in choice task *s* is given by

(3) $$\text{Prob}\left(V_{\mathrm{njs}}=1\left|X_{n1s}^{\prime },X_{n2s}^{\prime },\ldots ,X_{\mathrm{nKs}}^{\prime }\right),\Lambda \right)=\int \frac{\exp\left(X_{\mathrm{njs}}^{\prime }\beta \right)}{\sum_{k=1}^{K}\exp\left(X_{\mathrm{nks}}^{\prime }\beta \right)}f\left(\beta \left|\Lambda \right)\right)d\beta$$

where $X_{\mathrm{njs}}^{\prime }\beta$ are the attribute levels and the marginal utility parameters, and the vector Λ refers collectively to the parameters characterizing the distribution of the random parameters (e.g. mean and covariance of *β*), which the researcher can specify. Because the integral in Eq. (3) will not generally have a closed form, the probability can be approximated numerically through maximum simulated likelihood.

### 3.3 Selection of Attributes

Focus groups with farmers were conducted in Malawi's Central and Southern regions in July 2014 to identify the most important tradeoffs involved in annual versus perennial pigeon pea production. We also consulted with local agronomists and experts in legume cultivation in Malawi to define the parameters for the choice experiment. In the experimental design of the choice tasks we assume that pigeon pea is an intercrop with maize since arable land is scarce in the region and this is the most common practice with legumes in Malawi. The tradeoff between maize and pigeon pea yield was identified as a key factor in farmers' decision-making processes. This is mainly driven by the predominant role that maize plays in Malawian agriculture, diets and culture. Other factors identified as critical tradeoffs concerning annual versus perennial production of pigeon pea were the length of time the crop was in the field, the degree to which it improves soil quality, and the amount biomass produced. We model the hypothetical choice farmers face in the second year of a maize-pigeon pea intercrop, which is effectively the difference between an annual and perennial system. Modeling only the second year allows for direct comparison between the yield estimates of annual and perennial since respondents are evaluating the crop over the same time period.

The primary difference between an annual and perennial crop is the time the crop remains in the field. This translates into a lower input cost (seed and labor) since sowing is only required once a year but with a higher risk of crop failure (due to goat depredation, pest and disease, or extreme weather events). We communicate this complex concept as a binary choice by describing the logistics of producing a perennial crop (the crop remains in the field for either one season or over multiple seasons) without specifically calling it a perennial crop. Perennial crops tend to produce much higher levels of biomass since they have to devote less energy to root production after establishment. After ratooning,^1^ perennial pigeon pea produces a much higher level of biomass and we represent this as a dummy variable (low or high biomass production). Generally, low biomass is associated with an annual pigeon pea crop (e.g., a high harvest index) as opposed to the higher biomass associated with perennial crops since they have to devote less energy to root growth. Soil fertility is also included as a variable to capture the difference in soil fertility outcomes between an annual and perennial pigeon pea crop. Higher soil fertility is expected from a perennial variety since it has more extensive root growth and more time to recycle nitrogen.

The levels of the attributes were specified as what a farmer would get on average if they chose a particular option. Given the prevalence and importance of maize production in Malawi, we utilize maize yield as a proxy for a price or cost attribute. We do not present yield distributions because we found this to be a hard concept for farmers to understand. This attribute serves as a substitute for a cost or price variable when evaluating tradeoffs among the other attributes since maize is effectively a currency in Malawi. This indirect measure is preferable to a direct monetary variable, as many farmers are not able to accurately assess the monetary value of their output given the subsistence nature of agriculture in the region (Birol et al., 2009). The levels of the yield attributes were chosen to capture the trade-off farmers make when intercropping pigeon pea with maize. While there is variation among farms and study sites with regard to yields, area planted, and intensity of crop production, we set the amount of area under consideration to a finite amount of 0.5 acres.

According to Kamanga (2002) annual pigeon pea yields in Malawi range from 200 kg/ha to 600 kg/ha when intercropped with maize. Snapp et al. (2002) found yield of pigeon pea when intercropped with maize to range slightly higher from 380 to 650 kg/ha but these estimates were on research stations. We are not aware of empirical estimates of perennial pigeon pea yield so we based the levels for our attribute off of annual pigeon pea intercropped with maize. When grown as a perennial crop, pigeon pea yield can produce twice as much grain yield in the second year of harvest. In the experiment we used a range of pigeon pea yield from one to four 50 kg bags per half acre (a reasonable field size for farmers to consider), which is equivalent to about 250–1000 kg/ha (this roughly covers a lower bound estimate for annual to an upper bound estimate for perennial pigeon pea).

We used a range of maize yield estimates from three to six 50 kg bags of maize per half-acre or roughly 750 to 1500 kg/ha to roughly cover the range of subsequent year maize yields when intercropped with pigeon pea (continuous maize yield in Malawi is less than 2 tons per hectare on average). Note that this range is lower than the average yield per hectare for maize monoculture found in the literature since these estimates represent the second year of a perennial pigeon pea-maize intercrop which is hypothesized to be significantly lowered by increased competition. We converted estimates for pigeon pea and maize yields into acres for the choice experiments since that is the common unit of land measurement in Malawi.

### 3.4 Design of Choice Sets

There is a balance between the cognitive complexity of a choice experiment and the realism of the experiment (Louviere et al., 2000). This requires, among other things, selecting attributes or characteristics that are relevant to farmers' choices and conveying this information according to their cognitive abilities. We simplified yield data and fixed land area based on experimental data and communicated this in terms and measurements that farmers are accustomed to thinking about (50 kg sacs and acres). Our approach closely aligns with how agricultural extension officers present information on new crops to farmers.

**Fig. 2:**
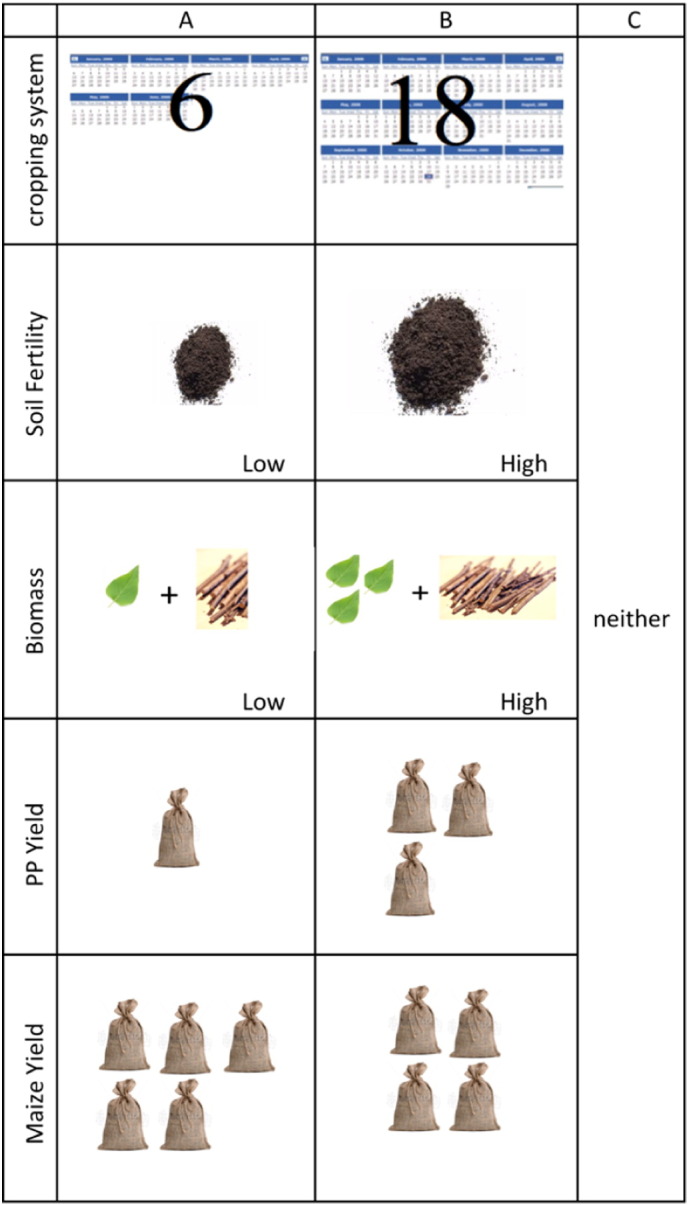
Sample choice task.

We utilized an orthogonal experimental design with the attributes and levels described above using Ngene software. A total of 40 choice tasks were generated and blocked into 8 groups of 5 choice scenarios. An option to opt out or select “neither” alternative was available to respondents during each choice task. Inclusion of a baseline or no choice alternative is important for the interpretation of respondent choices in terms of welfare economics and is consistent with demand theory (Louviere et al., 2000). We then created one illustrated booklet for each block, each containing 5 choice sets (see Fig. 2). To increase comprehension of the choice task, accommodate different farmer literacy levels and reduce the cognitive burden of this exercise, the choice tasks were illustrated and presented to farmers on laminated cards. The design was pre-tested to ensure that the tasks were relevant.

### 3.5 Data Collection and Questionnaire

The data were collected over a 3-week period beginning in October 2014 by trained enumerators. The questionnaire consisted of a series of questions about the socioeconomic status of the household (including household composition, education, assets, income), the farm characteristics (landholdings, crops grown, and respective harvest values), and the choice experiment. The questionnaire took approximately 1 h and we compensated each farmer with a one-kilogram bag of sugar for participation. The participants in the choice experiments were often the primary agricultural decision makers in the household.

### 3.6 Estimation

Our choice experiment approach allows for estimation of the tradeoffs farmers make when choosing to adopt a given cropping system. In choice experiment data analysis, estimation can be performed in either preference space or in willingness-to-pay (WTP) space. Coefficients obtained from models in preference space represent an individual's preferences or marginal utilities for the various attributes. The vector of parameters*??* defining preferences over the attributes can be interpreted as marginal utilities. The marginal rate of substitution (MRS) of one attribute for the other is simply the ratio of the two marginal utilities. For our purposes, we specify the coefficients corresponding to the attributes to vary, taking a normal distribution and also allowing for correlation of the random parameters. This allows for the possibility of positive and negative preferences for each of the crop system characteristics and a better understanding of the relationship between the attributes evaluated.

In addition to estimating our models in preference space, where we obtain marginal utilities, we also estimate this MRS tradeoff directly in willingness-to-pay space (Scarpa et al., 2008). Models estimated in WTP-space are reparameterized so that the coefficients estimated directly represent trade-offs individuals are willing to make; in this case the trade-off is calculated terms of maize yield. This approach facilitates direct control of the distribution of MRS estimates (as opposed to relying on the ratio of two marginal utility estimates with potentially undefined properties) and allows researchers to distinguish variation in preference (or MRS) versus scale heterogeneity.^2^

## 4 Results

### 4.1 Descriptive Statistics

Table 1 describes the households in our sample using basic summary statistics by region. Slightly over half of our respondents were females (58%), with significantly more women (73%) participating in our study in Zomba district. Our average respondent was 41 years of age with slightly over 6 years of formal education. Household size averaged approximately five persons across the three districts, with only minor variation from this mean. Households in our sample have been farming approximately 18 years, and farm size averages 2.4 acres or just under 1 ha, with modestly larger land holdings in Dedza and decreasing in size in the more southerly districts. Farmers in Ntcheu and Zomba have greater access to legume markets compared to those in Dedza, which is located in the central highlands. The majority of households used only their own labor with approximately 40% of households hiring outside farm laborers in the 2013–2014 growing season.

We asked farmers a series of questions related to their prior experiences with pigeon pea cultivation and use. Approximately 60% of the sample (292 farmers) had grown pigeon pea on their farm in the last 5 years (43 farmers in Dedza, 97 in Ntcheu, and 152 in Zomba). The majority of these farmers who cultivated pigeon pea planted a variety that matured between 3 and 6 months (85%). Of those that had grown pigeon pea, 28% reported having “ever ratooned pigeon pea” with 37% of them reported harvesting less or the same yield as annual pigeon pea and 63% reported harvesting more yield in subsequent years. Farmers also reported alternative uses of pigeon pea such as using it for fuel wood, soil amendment and forage (Table 2). Overall, the most widespread alternative use was as a soil amendment, and farmers in Zomba, where the most pigeon pea is grown, reported the highest rates of alternative uses of the pigeon pea plant (particularly as fuel wood).

Of those that grew pigeon pea, 44% reported some goat damage to their crop in the previous year. 83% of farmers reported this to be half of their crop or less. The goat damage was similarly distributed across the three districts.

### 4.2 Marginal Value of Perennial Pigeon Pea Attributes

Results from the choice experiments are displayed in Table 3. We present the results from the random parameters model specification allowing for correlation among variables (column 1).

The coefficient on the time the crop is in the field is positive and significant at the 1% level in the RPL model with correlation between random parameters. The soil fertility coefficient is large and twice as important to farmers as biomass production from pigeon pea. The coefficient on yield is positive for both pigeon pea and maize as expected but maize yield is valued by farmers approximately twice as much as pigeon pea yield. The coefficient on the opt-out variable is very large and significant indicating that many farmers gain more utility from not planting pigeon pea. The model fit statistics and the significance of the standard deviation estimates on the RPL model specification support the hypothesis of preference heterogeneity.

There is positive correlation between two attributes (Table 3a)—soil and time—implying that respondents who are motivated by increased soil fertility are also motivated by increased time in the field. The other attributes are not correlated at conventional levels of statistical significance. Since there are other attribute combinations where, for example, the amount of biomass produced by the crop is not related to the amount of soil fertility improvement, farmers would not necessarily have an association between these attributes.

The results from estimation in WTP-space model capture farmers' valuation of the cropping system attributes. Farmers are only willing to substitute a very small amount of maize (5% of yield) for a pigeon pea crop that takes 12 months longer in the field and this is only significant at the 10% level. They are willing to substitute a larger portion of their maize yield (36.5%) for a higher level of soil fertility and willing to substitute 16.3% of their maize yield to reach a higher level of biomass production. Farmers are only willing to trade a 0.61% percent increase in pigeon pea yield for a 1% increase in maize yield.

### 4.3 Demand for Perenniality

**Fig. 3:**
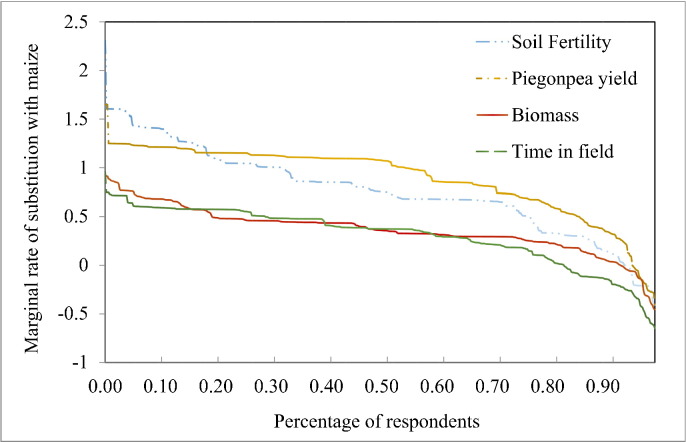
Demand for attributes of a perennial pigeon pea-maize intercrop.

We illustrate the demand for “perenniality” as a function of the demand for each of the individual attributes associated with the production of a perennial crop. A perennial pigeon pea crop remains in the field for a longer period of time (18 months), has higher soil fertility, higher biomass production, and more pigeon pea yield. Fig. 3 below depicts the relative demand for each of the four attributes in terms of maize yield.

Demand for perenniality is generally positive (for about 85% of the sample) but farmers place a very low value on it due to the interplay of various tradeoffs associated with perennial pigeon pea production. Even within a single variable such as the amount of time the crop is in the field there can be tradeoffs such as the lower labor requirements associated with perenniality along with higher risk of crop failure since the crop is in the field longer. Biomass is only marginally more appealing and follows a similar demand curve. Soil fertility improvement is a highly valued attribute to farmers dominating biomass production and perenniality across the sample. The curve is steeper than the other attributes initially indicating that a portion of the sample (lower 15%) would trade a very large maize yield loss for soil fertility improvement. Demand for pigeon pea yield is also an important attribute for farmers in a maize-pigeon pea intercrop but more elastic than demand for soil fertility and relatively consistent across the sample.

### 4.4 District Level Differences

We also estimated RPL models for each of the districts individually allowing for correlation of attributes. In Dedza and Ntcheu time in the field is only a marginally significant determinant of choice. The significance of the standard deviation coefficients on time in the field suggests that there is a subset of the population in these districts that has a higher value for this attribute. Soil fertility is still the most important attribute to farmers at the district level, with the largest marginal utility in Zomba. The coefficient on biomass is also significant in all districts but is much less important in Ntcheu than Dedza and Zomba. The coefficients on pigeon pea yield and maize yield are significant in all districts. Pigeon pea yield has the highest marginal utility in Zomba with the least amount of variation. Maize yield is also highest in Zomba and there is significant heterogeneity in preferences for maize yield across the three districts. (See Table 4).

Similar to the overall model, there is a relatively strong correlation between time in the field and soil fertility in Ntcheu (negative) and Zomba (positive). In Dedza there is a correlation between time in the field and biomass production. And in Zomba there is a positive correlation between pigeon pea yield and biomass production. (See Tables 4a, 4b, and 4c).

### 4.5 Farmers Who Opted Out

Finally, we compare the respondents who selected one of the change alternatives in a given choice task (Opt-in) with those that opted out (Opt-out). Overall, farmers opted out of a relatively large portion of scenarios, not choosing either of the options involving pigeon pea-maize rotations (69%, 69%, and 68% in Dedza, Ntcheu, and Zomba respectively). We tested the difference in the mean across a set of variables of respondents who opted in or opted out of the choice altogether. The percentage of people that opted-in was almost identically distributed across the three districts, meaning that the population of people that opted out was similarly distributed across the districts as those that opted in. None of the demographic or socioeconomic variables were significantly different between respondents that chose a maize-pigeon pea scenario and those that opted out. There were significant differences between the percentages of respondents in each of the food security categories within each group, however there were no statistical differences between the percentage of respondents in each category between the two groups. (See Table 5).

## 5 Discussion

While pigeon pea is cultivated widely in Southern Malawi where there are robust markets for pigeon pea, there is limited production in the central regions. It is logical that there would be numerous scenarios where farmers obtain greater utility from choosing to opt out of the choices that included pigeon pea since many of them are not familiar with pigeon pea and are not as interested in intercropping with pigeon pea as other leguminous crops (Ortega et al., 2016). The tradeoff associated with perennial cultivation, including lower labor costs but more risk exposure through a longer time in the field is a non-negative attribute for most farmers but not significantly positive on average. Farmers place higher value for improving soil fertility and they value it almost twice as much as the biomass they obtain from the crop.

Respondents in Dedza reported the lowest rates of education and wealth, are the furthest from markets and have the largest land holding size, and rent in the most labor. These characteristics are all amenable to perennial production and suggest more of a subsistence production strategy and less intensive agricultural production. These farmers could benefit from the labor reduction of a perennial crop and are the least able to afford inputs for more intensive production so would also benefit from the soil fertility improvement. At the other end of the spectrum is Zomba district with the highest population density, the smallest average land holding size, the closest reported proximity to markets (especially pigeon pea markets), and arguably more intensive agricultural production (most hybrid maize production and higher off farm income). It makes sense that farmers in Zomba would be the most interested in both soil fertility improvement and grain yield of both maize and pigeon pea for this reason. This is particularly true of pigeon pea yield, where the marginal utility is highest since this is a cash crop for many farmers in Zomba. In Dedza, the expectations of maize yield are lower but there is a much smaller standard deviation around the estimates suggesting that more farmers in Dedza would be willing to give up maize yield, presumably for perenniality.

There are two possible interpretations of the low demand for perennial pigeon pea. One interpretation is that there is little market demand for perennial pigeon pea and this is largely driven by low overall demand for (annual) pigeon pea. Another interpretation, which focuses more on underlying behavioral dynamics, is to look at the results in terms of the theoretical benefits of perenniality. The demand for the ecological services associated with perennial pigeon pea is not well developed. Improving extension efforts and educating farmers on the benefits of perenniality could help overcome this barrier to adoption. It is possible that perennial cultivation of pigeon pea would be more popular than annual cultivation of pigeon pea if perennial varieties were developed and agronomic recommendations made available that promoted multiple harvests.

Given the stronger preferences for soil fertility and pigeon pea-maize yield attributes in Zomba it is likely that these farmers are evaluating the choices as a cash crop producer. Farmers in Dedza, which tend to have less assets and have less access to markets appear to be more open to perenniality. A logical conclusion is that there may be a niche for perennial pigeon pea in some regions while in other regions shorter duration higher yielding varieties would be preferred.

The finding that biomass has a positive and significant value is generally consistent with a recent study in Southern Malawi, which explored the impact of adoption of new variety of pigeon pea with thick stems, ‘*Mthawajuni*’ (Orr et al., 2014). The authors make the case that even though the new variety had superior fuel wood traits, grain production was still the most important attribute to farmers. This appears however, to be a very minor driver of demand for pigeon pea compared to the other relevant attributes which we consider.

## 6 Conclusions

While perennial crops have numerous ecological and agronomic advantages over their annual counterparts, they still face significant barriers to adoption by smallholder farmers. In some instances, a crop variety bred explicitly for perennial production qualities may be more appealing to farmers than a variety bred for annual qualities due to some of the ancillary benefits associated with perenniality. While some farmers view the primary tradeoff associated with perennial pigeon pea intercropped with maize to be competition with maize in subsequent years of production, others appear to see synergies between pigeon pea and maize yield. While maize yield in a maize-legume intercrop is of paramount importance to Malawian farmers, we find positive yet heterogeneous demand for pigeon pea perenniality driven by high demand for both soil fertility improvement and pigeon pea grain yield.

These findings have implications for current agricultural policy in Malawi, the cornerstone of which is the Farmer Input Support Program (FISP). Chibwana et al. (2012) found that FISP increases land area planted to improved maize and tobacco at the expense of traditional maize and crop diversity including legumes. Given the heterogeneity in preferences for the ancillary benefits of perennial and legume crops, a maize subsidy program like FISP will negatively impact soil fertility in the long run for many farmers in Malawi. There is also evidence that FISP is less likely to reach asset poor households (Ricker-Gilbert et al., 2011). These asset poor households are the households most likely to adopt low cost sustainable technologies like perennial pigeon pea and the households that would benefit the most from soil fertility improvement through perennial and leguminous crops. If FISP was able to broaden its scope to include leguminous crops it would be better able to support the poorest households.

Additional research should explore the components of perennial production by further deconstructing attributes of perenniality such as the value of the labor and seed input reduction. Exploring the relationship of farmers' risk preferences to their cropping choices would allow us to understand if more risk-averse farmers are likely to adopt low input technologies such as a perennial pigeon pea crop. Another possible research area is to explore demand for a combination of traits such as short duration (e.g., earlier flowering period) with perennial production to minimize labor costs while minimizing the risk of yield loss from depredation.

## Figures and Tables

**Table 1:** Summary statistics of farmers in sample.

Characteristic	Dedza	Ntcheu	Zomba	Total
Household size (persons)	5.25	5.06	4.88	5.07
Female (%)	49%	54%	73%	58%
Age of respondent (years)	40.27	42.72	39.06	40.70
Education (years)	4.94	7.00	6.41	6.12
Under 16 (persons)	2.45	2.32	2.35	2.38
Years farming	17.99	19.34	17.45	18.27
Landholding size (acres)	2.79	2.27	2.03	2.37
Distance to nearest market (km)	7.1	4.62	4.22	5.32
HH labor (previous yr. in persons)	3.05	3.18	3.06	3.09
Hired labor (% reporting)	43%	34%	40%	39%
N	162	165	161	488

**Table 2:** Importance of pigeon pea attributes (other than grain yield) by district.

	Dedza	Ntcheu	Zomba
Fuelwood	44%	80%	95%
Soil amendment	74%	85%	89%
Forage	40%	38%	51%
N	43	97	152

**Table 3:** Random parameter model results.

	Preference space		WTP-space	
	Coefficient	Std. error	Coefficient	Std. error
*Random parameter means*				
Time in field	0.164***	0.056	0.050*	0.025
Soil fertility	0.768***	0.074	0.365***	0.029
Biomass	0.358***	0.057	0.163***	0.026
Pigeonpea yield	0.015***	0.001	0.611***	0.054
Maize yield	0.023***	0.001	–	–
Opt-out	2.110***	0.358	2.610***	0.306

*Random parameter standard deviations*				
Time in field	0.347***	0.106	0.182***	0.042
Soil	0.660***	0.103	0.316***	0.037
Biomass	0.517***	0.094	0.228***	0.034
Pigeonpea yield	0.008***	0.002	0.252**	0.109
Maize yield	0.010***	0.002	–	–

*Model fit statistics*				
N		2440		2440
Log-likelihood		− 1534.296		− 1379.071
AIC		3080.6		2780

Note: ***, **, * represent significance at the 1%, 5%, and 10% levels. Random parameters logit model estimated using NLOGIT 5.0 based on 1000 Halton draws used for simulated maximum likelihood.

**Table 3a:** Correlation matrix for RPL model in Table 3.

Correlation matrix	1	2	3	4	5
Time in field (1)	1.000	0.998	0.147	0.515	0.061
Soil (2)		1.000	0.143	0.465	0.006
Biomass (3)			1.000	0.009	0.090
Pigeonpea yield (4)				1.000	0.426
Maize yield (5)					1.000

**Table 4:** Random parameter model results by district.

	Dedza		Ntcheu		Zomba	
	Coefficient	Std. error	Coefficient	Std. error	Coefficient	Std. error
*Random parameter means*						
Time in field	0.165*	0.099	0.175*	0.103	0.086	0.101
Soil fertility	0.725***	0.139	0.773***	0.122	0.995***	0.158
Biomass	0.440***	0.102	0.260**	0.110	0.409***	0.111
Pigeonpea yield	0.014***	0.002	0.015***	0.002	0.016***	0.002
Maize yield	0.022***	0.002	0.024***	0.003	0.027***	0.003
Opt-out	2.056***	0.589	2.823***	0.619	2.071***	0.785

*Random parameter standard deviations*						
Time in field	0.522***	0.178	0.580***	0.160	0.257	0.177
Soil fertility	0.759***	0.188	0.630***	0.168	0.720***	0.176
Biomass	0.343**	0.151	0.693***	0.202	0.595**	0.233
Pigeonpea yield	0.011***	0.003	0.009***	0.003	0.008**	0.004
Maize yield	0.009***	0.003	0.011***	0.002	0.011**	0.004

*Model fit statistics*						
N		810		825		805
Log-Likelihood		− 465.148		− 489.307		− 393.75
AIC		972.3		1020.6		829.5

Note: ***, **, * represent significance at the 1%, 5%, and 10% levels. Random parameters logit model estimated using NLOGIT 5.0 based on 1000 Halton draws used for simulated maximum likelihood.

**Table 4a:** Correlation matrix for Dedza.

Correlation matrix	1	2	3	4	5
Time in field (1)	1.000	− 0.508	0.723	− 0.171	− 0.237
Soil (2)		1.000	− 0.658	− 0.547	− 0.180
Biomass (3)			1.000	0.171	0.306
Pigeonpea yield (4)				1.000	− 0.042
Maize yield (5)					1.000

**Table 4b:** Correlation matrix for Ntcheu.

Correlation matrix	1	2	3	4	5
Time in field (1)	1.000	− 0.942	− 0.393	− 0.235	0.052
Soil (2)		1.000	0.609	0.121	− 0.069
Biomass (3)			1.000	0.293	− 0.013
Pigeonpea yield (4)				1.000	0.209
Maize yield (5)					1.000

**Table 4c:** Correlation matrix for Zomba.

Correlation matrix	1	2	3	4	5
Time in field (1)	1.000	0.984	0.481	0.390	− 0.422
Soil (2)		1.000	0.407	0.246	− 0.534
Biomass (3)			1.000	0.773	0.357
Pigeonpea yield (4)				1.000	0.611
Maize yield (5)					1.000

**Table 5:** Profiles of farm households that opted-in and out of the choice tasks.

Variable	Opt-in	Opt-out	*p*-Value
District			
Dedza	33%	33%	0.95
Ntcheu	33%	35%	0.67
Zomba	34%	32%	0.72
Male	45%	38%	0.12
Age of respondent (years)	40.68	40.72	0.99
	(0.83)	(0.91)	
Education (years)	5.90	6.37	0.31
	(0.20)	(0.36)	
Landholding size (ha)	2.40	2.39	0.76
	(0.10)	(0.09)	
HH labor (previous year in persons)	3.10	3.08	0.78
	(0.09)	(3.35)	
Extension visits (no. per year)	3.70	3.35	0.34
	(0.30)	(0.25)	
Maize area (acres)	0.61	0.62	0.88
	(0.05)	(0.06)	
Food security			
Shortage throughout	1%	0%	0.01
Occasional food shortage	23%	15%	
No shortage or surplus	24%	21%	
Surplus	5%	9%	
Observations (N)	261	267	

Note: Numbers in parenthesis are standard deviations; *p*-values presented are for joint tests of significance (Chi-squared or Mann Whitney).

## Footnotes

1 Ratooning is removing the leaves of a crop and leaving the roots and the lower parts of the plant so the crop that emerges the following season matures earlier.

2 Interested readers are pointed to Scarpa et al. (2008) and Sonnier et al. (2007) for a more in-depth discussion of the advantages of estimating choice models in WTP-space.
